# Towards an informed research agenda for the field of personality disorders by experts with lived and living experience and researchers

**DOI:** 10.1186/s40479-024-00257-0

**Published:** 2024-07-08

**Authors:** Babette Renneberg, Joost Hutsebaut, Ann Berens, Chiara De Panfilis, Katja Bertsch, Andres Kaera, Ueli Kramer, Christian Schmahl, Michaela Swales, Svenja Taubner, Mariana Mendoza Alvarez, Julia Sieg, 30 experts with lived experience – clients, relatives, significant others - from 10 European countries

**Affiliations:** 1 Department of Clinical Psychology and Psychotherapy, Freie Universität Berlin, Habelschwerdter Allee 45, 14195 Berlin, Germany; 2 German Center for Mental Health (DZPG), partner site Berlin, Berlin, Germany; 3 Department of Medical and Clinical Psychology, Tilburg School of Social and Behavioral Sciences, Tilburg University, Tilburg, The Netherlands; 4 Scientific Initiative of Neuropsychiatric and Psychopharmacological Studies (SINAPS), University Psychiatric Centre Duffel, Duffel, Belgium; 5 Collaborative Antwerp Psychiatric Research Institute, Universiteit Antwerp, Antwerp, Belgium; 6 Department of Medicine and Surgery, Unit of Neuroscience, University of Parma, Parma, Italy; 7 Department of Psychology, Julius-Maximilians-Universität Würzburg, Würzburg, Germany; 8 Department of Psychiatry, Kanta-Häme Central Hospital, Wellbeing Services County of Kanta-Häme, Hämeenlinna, Finland; 9 Department of Psychiatry, Institute of Psychotherapy and General Psychiatry Service, Université de Lausanne, Lausanne, Switzerland; 10 Department of Psychosomatic Medicine, Medical Faculty Mannheim, Central Institute of Mental Health, Heidelberg University, Heidelberg, Germany; 11 German Center for Mental Health (DZPG), partner site Mannheim, Mannheim, Germany; 12 North Wales Clinical Psychology Programme, Bangor University, Bangor, Wales; 13 Institute for Psychosocial Prevention, Ruprecht-Karls-Universität Heidelberg, Heidelberg, Germany; 14 Faculty of Medicine and Health Sciences, Collaborative Antwerp Psychiatric Research Institute (CAPRI), University of Antwerp, Antwerp, Belgium

**Keywords:** Public and patient involvement, Experts with lived experience, Personality disorder

## Abstract

**Background:**

We describe a collection of themes for a research agenda for personality disorders that was originally formulated for the ESSPD Borderline Congress in 2022.

**Methods:**

Experts with lived and living experience (EE), researchers and clinicians met virtually, exchanged ideas and discussed research topics for the field of personality disorders. The experts - patients, relatives, significant others - named the topics they thought most relevant for further research in the field. These topics were presented at the ESPPD conference in October 2022.

**Results:**

The five top themes were: 1. Prevention, early detection and intervention, 2. Recovery beyond symptom improvement, 3. Involvement of relatives in treatment, 4. Gender dysphoria, and 5. Stigma.

**Conclusions:**

In general, the topics reflect current issues and changes in societal values. Overarching aims of research on these topics are the improvement of social participation and integration in society, better dissemination of research, and better information of the general public and political stakeholders.

## Introduction

The importance of the perspectives of service users in research on mental health has been increasingly recognized over the last decades. While historically, experts with lived and living experience and their significant others (EE) were primarily seen as the object of clinical and research endeavors, there is a growing number of projects and attempts to create collaborations and partnership between experts by profession and EE. This participatory approach is also called public and patient involvement [11]. To improve this situation, many research funding agencies now require EE to be involved in research. Ideally, this should be the case in every step of the research. As Lloyd and White [32] pointed out: Research priorities are rarely set democratically. Priorities for research of academic researchers differ from those of EE.

An exemplary approach in this field is established by the James Lind Alliance (JLA https://www.jla.nihr.ac.uk/) Priority Setting Partnerships in Oxford, UK. The JLA was established in 2004 and is funded by the UK Medical Research Council and National Institute for Health Research (NIHR). This non-profit initiative brings together patients, carers and clinicians to identify and prioritize research questions that should be further explored and funded for a given disease or disorder. The aim of such initiatives (see also e.g., [2]) is to integrate patients and relatives as co-creators in all stages of the research process. Ethical and legal reasons form the base for this participatory process which also ensures that research becomes more relevant and more credible for those affected by the disease or the disorder.

However, PPI has hardly been realized in mainstream psychological/psychiatric research in many countries, especially when it is accompanied by actual decision-making power of EE. The reasons for this include that PPI is cost- and time-intensive, sometimes it is difficult to find EE partners, and that power over the research process is reluctantly shared [11]. Furthermore, in many countries an organized structure for PPI does not exist. Thus, it is difficult to find EEs for researchers if structures from funding agencies are not yet established and no funds exist to pay the effort and the participation of EE.

Recently, a growing body of research involving EE has emerged. Here, a distinction should be made between research that seeks to explore the experiences and perspectives of EE (e.g., [3, 48]), and research that is planned and conducted *in collaboration with* EE (e.g., [14], see below). While the former reinforces the patient – practitioner dichotomy, the latter creates the potential for new forms of equal partnership. One example of such collaborative research is a qualitative study on understandings of recovery [14]. In this study, the research team consisted of one service user researcher, one university researcher and a clinical researcher. In an additional article, the authors reflect upon their work process and the way their different perspectives enhanced and challenged the collaboration [13]. Obviously, an equal partnership does not happen easily and different perspectives demand more reflection and dialogue than traditional research teams might face. But the approach also holds the potential for the team to widen the perspective, yielding in more relevant research and results.

Involvement of EE matters for multiple reasons and at different levels. Priorities change depending on whether the priorities of EE and experts by profession tend to differ. A vivid example is provided by Adebajo who reflected upon his experience of participating in two James Lind partnerships – first as a stroke patient and then as a medical professional [1]. He himself seemed surprised how his priorities differed in the two partnership settings. As a medical professional, he was favoring pathogenetic, diagnostic and treatment priorities. When he participated as a patient, however, he chose support and rehabilitation priorities instead – a pattern that has been observed before [1].

Another reason for the involvement of experts with lived experience (EE) in research is the empowerment and the potential reduction of stigma that comes through the process of being actively engaged in research. It should be noted that in research on personality disorders it is sometimes difficult to engage EE in the process for various reasons, among them the fear of speaking openly in public. Often, people with a personality disorder and their family members have experienced a long history of discrimination and have difficult treatment experiences within the mental health system (e.g., [34], for family members see [30]). This issue is reported across various professions (e.g. [26]) and is paralleled by the finding that mental health professionals often report rather pessimistic attitudes toward patients with personality disorders, who might still today be regarded as “the patients psychiatrists dislike” [28]. The process of being actively engaged in research could be an experience that is empowering and has the potential to reduce stigma.

In line with the experience of Adebajo [1], a first step to involve EE might be to collect research priorities from EE and their relatives. Thus, we set out to identify which research questions EE prioritize with the aim to create a starting ground for more collaborative research in the field of personality disorders. Here, we report on the outcomes of this initiative and complement with some additional comments that were raised during the subsequent discussion during the presidential debate of the 2022 ESSPD congress, where the findings were presented.

## Methods

Aim of the study was to identify research questions of EE to create a starting point for more collaborative research in the field of personality disorders.

### Procedure

The research agenda was developed in two rounds. In the first round, we collected input from several Dutch and Belgian EE and family associations. These countries were chosen as the starting point, because originally the plan was to have the ESSPD congress in Antwerp, Belgium. EE and representatives of EE associations were asked what topics for research they considered to be the most important in the field of PDs and what specific research questions they would like to see addressed by the research field. Input was received in two ways: by sending out a survey to all major patient and family societies in Belgium and the Netherlands (in the field of PDs and broader) and by organizing a focus group with representatives of these societies. EE were encouraged to 1) formulate research topics and questions they considered to be of interest from their perspectives and 2) to argue why they found these topics and questions to be important to them. Based on their input, overarching themes were identified by two authors (J.H. and A.B.), including a large number of specific questions. The resulting proposal including research themes, their description, and their rationale were sent by email to the Belgian and Dutch participants to obtain agreement. In a second round, participants from various European countries provided additional comments and suggestions to this proposal, in different ways (i.e., some of them wrote extensive comments, others marked the various themes / questions in order or relevance). The input of these international participants was then added in the definitive proposal, with the goal to be as inclusive as possible in representing the perspective of people with lived experience across all Europe. No specific consensus scores were calculated.

### Participants

Participants for the first round were recruited through Belgian and Dutch patient and family societies. We contacted them, explained our goal related to the upcoming conference and asked for their participation. Representatives from five societies were involved in the inventory, and most of them retrieved ideas from peer-EE within their societies. Participants for the second round were recruited through the ESSPD board. Board members reached out to EE societies or individual EE from various European countries, either personally or by asking colleagues in their professional networks to spread the notice of this initiative among EE they were in contact with. Both, individual consumers or carers/family members with lived/living experience and representatives of EE societies were invited to participate. This was accomplished by sending them an email detailing the background and scope of the initiative. Participants were asked to provide their ideas and feedback upon the input from the first round, indicate their priorities and suggest additional themes or edits / rephrase some research questions. Potential respondents were informed that this inventory represented a first effort to present the input and expertise of EE to the research community at the Opening Ceremony of the 2022 ESSPD Conference. In total, more than 30 persons with lived experience and relatives from nine European countries, including Belgium, Sweden, Denmark, Norway, Spain, Germany, Italy, the Netherlands and Switzerland, provided input for the research agenda. Overall, the idea of involving clients and relatives in research was very well received. Some respondents expressed their hope to continue this work.

In Table 1 questions as submitted by EE are listed. These questions were grouped into topics and summarized by professional experts (J.H. and B.R.).

**Table 1:** Topics and possible research questions

**Topic**	**research questions**
1.Prevention early detection and intervention	• What is needed for personality disorders to be diagnosed early on so that earlier appropriate treatment is possible? • What measures can be taken to help parents and schools, as well as public health institutions, detect a developing personality disorder? • Specific to Cluster C PDs: how can clients be helped to take their own burden seriously and seek appropriate treatment? • What protective elements in early development help a personality develop in a healthy way and can be delivered through schools, for example? • How should mental health services be set up so that clients can get appropriate help within a reasonable timeframe? • What are advantages and disadvantages of a diagnosis of PD for young people? In other words: could the advantages of an early diagnosis, which can lead to early interventions for clients and families, be more important than the potential disadvantages correlated with stigma? • What initiatives can lower the threshold to seek help? • Can free, digital psycho-educational interventions help at an early stage?
2. Recovery beyond symptom improvement	• Which treatments really help to recover social functioning? Do comparative studies on this topic exist? • What kind or level of meaningfulness is achieved after treatment? At what point during or after treatment are these forms of meaningfulness achieved? • How can treatment programs collaborate better with local, community-based social institutions focused on recovery (career support, volunteering, recovery initiatives)? • Which work environments are best suited to the vulnerabilities of people with personality disorder (think of opportunities for flexible work in terms of hours, opportunities for work from home)? • How can treatment better align (in terms of outcomes) with a specific client's needs rather than using general measures that are assumed to be desirable for everyone? • How can you help clients prevent relapse? How can clients learn to distinguish relapses from normal dips? How can clients learn to better manage relapses without having to start new treatments? • How can treatment improve inclusion in society for those clients whose social life is severely impaired? • Can the provision of specific guidelines about healthy lifestyle and the inclusion of the family in the follow-up help to prevent relapse?
3. Involvement of relatives in treatment	• Does it add value to involve family members and other loved ones? How can clients be better informed about the potential added value? • What skills of loved ones contribute to better client recovery? • What skills of loved ones contribute to a better relationship with the person with (borderline) personality disorder? • What information for loved ones might be helpful so that they can better relate to the client with a personality disorder? • What strategies help loved ones to recognize and reduce the client's suicidality? How do reactions of loved ones affect a crisis and which reactions are best? • Can support for loved ones and psychoeducation contribute to improved well-being? • Can loved ones help motivate clients who, because of the nature and severity of their personality disorder, tend to avoid care or discontinue treatments early? • Can experts by experience add value to treatment and how should they be used? • Can informal networks (peers) contribute to recovery? • How does the cultural context around the client differ and how can counselling better fit the existing cultural context? • What interventions are needed with children of parents with personality disorders? What if parents are suicidal or self-harming? • Can self-help groups for families be useful? • Can a separation from a disturbed family environment improve the future life of the young patient with PD? • Should the system of treatment be better tailored to the patient and follow the patient instead of the other way around?
4. Gender dysphoria	• How is the association between gender dysphoria and personality disorders? • Does gender transition put people with personality disorders at risk or is it just appropriate when gender dysphoria is present?
5. Stigma	• How to decide whether or not to disclose about personality disorder diagnosis? • How can people acquire a new identity after treatment where they can leave their 'label identity' behind? • Is there indeed a greater stigma attached to the diagnosis of personality disorders than to other diagnoses? • Does the degree of stigma people experience also depend on how the diagnosis was given and/or the treatment instituted following the diagnosis? • Can making the diagnosis reinforce clients' self-stigma or does it just defuse it? Does the 'label' add value? Can focusing too much on the diagnosis lead to “live with the illness”, to the extent that the illness becomes the “cornerstone” of one’s own life? • Could it be that the stigma also leads to misdiagnoses, such as Autism Spectrum Disorders, which may be less stigmatizing?

## Results

The final consensus proposal consisted of five research topics for personality disorders that were considered most important by 30 EE:

1. Prevention, early detection and intervention
2. Recovery beyond symptom improvement
3. Involving relatives in treatment
4. Gender dysphoria
5. Stigma

In the following, we will briefly summarize the reasons why each theme was considered important according to and approved by participants and which specific research questions arose (see Table 1). The first three themes were mentioned as the most important ones, in this order. In the discussion section of this paper, each theme is elaborated further.

### Prevention and early detection and intervention

Almost all EE recognize that they or their affected family members were diagnosed and treated for their personality disorder at a late stage. Many EE live their daily lives for years with symptoms that severely impair their functioning. EE think that a delayed diagnosis conditioned their life and their decisions. Notably, many EE explicitly mention that they personally do not consider a personality disorder diagnosis stigmatizing (see also 5. Stigma). Rather, they report that receiving a diagnosis not only grants them access to appropriate care, but also provides an explanation about the way they feel, they deal with the world, and ultimately makes them feel less alone – “there are others like us”. Furthermore, calling the personality disorder “by its name” gives meaning to the suffering of the affected individuals, and lowers their feelings of inadequacy. EE also pointed out the need for the mental health system to communicate and to collaborate with other services, e.g., community services in order to facilitate early diagnosis (i.e., schools, GPs…). Regarding preventive measures, the question was raised: What protective elements in early development help a personality develop in a healthy way and can be delivered through schools, for example? For other specific research questions see Table 1.

### Recovery beyond symptom improvement

Research mainly evaluates whether treatments lead to an improvement of symptoms. For many EE, this does not necessarily imply that patients can actually participate in society (again). Many clients find it difficult to find a new meaning in life and a new identity after treatment. Thus, recovery and integration in social life (i.e., job, relationship) is vital from the perspective of both clients and their relatives. EE think that multiple factors affect recovery, and these factors need to be ascertained on an individual basis. They also think that full recovery implies that an individual is integrated into one’s own social environment, in accordance with his/her attitudes and dispositions. For specific research questions see Table 1.

### Potential benefits of involving relatives in treatment

Personality disorders have an impact on relationships. Family members and friends often find it difficult to assess what they should or should not do. The family/partner relationship can also be inhibitory to the client's recovery. EE have the impression that in many cases it could be positive for treatment if family members were more systematically involved in treatment. This is particularly true when the client him-/herself tends to avoid or reject care or repeatedly terminates treatment prematurely. In addition, there are several other forms of support besides professional care that could be helpful, e.g., self-help groups. For specific research questions on this topic see Table 1.

### Gender dysphoria

This is a specific theme that recurred repeatedly among EE. Questions related to gender identity seem to have become much more common in recent years. It can be unclear whether the experienced gender dysphoria is part of the personality disorder, a comorbid disorder or unrelated. E.g., relatives have the impression that there is a link with autism, but possibly also with personality disorders. Moreover, it raises the question of what the risks or benefits of a medical course of transition might be. For specific research questions on this topic see Table 1.

### Stigma

The statement of many clients that they personally do not consider a personality disorder diagnosis stigmatizing may be somewhat surprising because there is the assumption that a diagnosis of a personality disorder carries a strong societal stigma. This is particularly true for the diagnosis of borderline personality disorder (BPD). EE struggle with the pros and cons of offering openness about the diagnosis, for example to employers or to other medical doctors. They suggest that, ideally, longitudinal research evaluating the advantages of an early diagnosis in terms of access to and effectiveness of individual and family interventions could clarify whether this approach is superior to the potential harm conveyed by stigma. For specific research questions see Table 1.

## Discussion

Our aim was to inform which research topics experts with lived experience (EE) regard as important for the field of personality disorders.

Before discussing the results in more depth, we wish to highlight how important it is that researchers and clinicians listen to the EE’s attitudes, preferences, and values and consider them in the research process. To illustrate the point: EE may have a different definition of recovery.

The emergence of *early detection and intervention* as a number one priority from the perspective of EE is remarkable in the light of a longstanding reluctance to diagnose personality disorders at an early stage [6]. Whereas most professionals used to delay the diagnosis [27], based upon a range of misconceptions [44], EE prioritize the need for early detection and intervention strategies in research. Early intervention approaches for PD, particularly borderline personality disorder (BPD), have been developed, with numerous programs now available for young individuals diagnosed with BPD [8] . Specifically, at least five early intervention programs have been established in Australia, The Netherlands, and Germany [7, 19, 22, 24, 43]. Additionally, programs in Norway and the UK predominantly focus on addressing self-harm behaviors [33, 42]. Main critiques of existing literature highlight several key issues, hampering the integration of early intervention for PD within mainstream health services: 1) stigma and discrimination, 2) lack of trials prioritizing adaptive functioning as main outcome, 3) trials have not adequately included emerging adults with early-stage PD, and 4) the need to clarify the complexity of treatments like general community care (GCC) and enhanced usual care (EUC) what makes them efficient and role of individual therapy [8].

Interestingly, the issue of stigma was also raised by EE in the context of early detection and notably, EE favored research studying the advantages of early detection as opposed to potentially stigmatizing effects. Several EE spontaneously mentioned that they had faced stigmatizing and sometimes punitive reactions from caregivers and a lack of compassionate understanding due to their dysregulated behavior. An early diagnosis that is comprehensively explained [31] might disrupt trajectories of long-term impairment. Albeit this research is on the rise, there is still much to be done to explore how to best help adolescents with BPD. This emerging field of interventions for adolescents should continue. Research should address this longstanding reluctance by including patients’ perspectives, especially highlighting the advantages of an early diagnosis over those conveyed by potential stigma. According to the EE involved in this research, it may actually be more stigmatizing not to diagnose early. However, note that this issue is discussed very controversially in the field [17].

The second theme touches upon the core of what it means to *recover* from a personality disorder. For BPD, most psychotherapeutic treatment studies show promising results [45], but treatment effectiveness is often restricted to the improvement of symptoms of personality disorders. In a meta-analysis, Zahediabghari et al [49] summarize that specifically-designed psychotherapies for patients with BPD can improve psychosocial functioning more than unspecific psychotherapies.

However, EE point to the importance of social reintegration and discovering meaningfulness in life [36]. Several clients mentioned they had followed more than one personality disorder-oriented treatment, which had been very helpful, but who felt they were still struggling with full recovery and satisfactory inclusion in society. Some research is available on this topic and points in the same direction: In a qualitative study, Gillard, Turner & Neffgen [14] examined the understanding of recovery in the context of lived experience with personality disorders. The authors conclude that key facilitators of recovery were positive personal relationships and wider social interaction. Similarly, self-generated treatment goals by 102 patients with BPD showed that while patients value symptom improvement, 88 % also wanted better psychosocial functioning, including better social relationships and a job [34]. Patients with BPD also emphasize that personal recovery is represented by practical achievements in the “capacity to work and love”, as indicated by three key themes: 1) love of self and others, 2) making a contribution through work and study, 3) stability in daily life [16]. These results are comparable to those of qualitative studies focusing on work-related functioning (e.g., [29, 35]).

The need for integrative care emerged as the central topic. From her lived experience, one of the participants recalled that not only psychotherapy helped her, but a whole network of people who supported and respected her. It was in the togetherness with others, she said, that she was able to be herself and to feel connected. Although BPD is associated with problems in social connectedness, it remains uncertain if these social challenges reciprocally worsen or trigger BPD symptoms. To clarify this potential bidirectional relationship, further longitudinal studies are necessary [10]. For further research, we recommend to include measures of recovery of psychosocial functioning next to symptom improvement in treatment outcome studies. Furthermore, the field might benefit from more insight into what “psychosocial (dys)function” means in personality disorders.

Regarding the third theme, a diagnosis of a personality disorder affects not only the individual, but also *family members* and others at school and at the workplace. The wider network could become a valuable resource for recovery if support and psycho-education about personality disorders are provided. Some psychoeducation programs for carers of patients with BPD have been found to be effective in improving their communication skills toward their affected family member, their psychological well-being and their knowledge of the disorder and in decreasing their subjective burden [15, 20, 21, 38, 39]. However, most research is focused on BPD only and evaluates the effects of psychoeducation on family members only. The focus of the research should also include evaluations to reveal if the training, psycho-education or involvement in treatment for family members has a measurable, beneficial impact on their loved ones. For clinical practices, one recommendation could be to include context-based and systemic interventions as part of treatment for personality disorders (e.g., family interventions). While this is usually done in treatment of youth with personality disorders (e.g. [42]), treatments for adults usually lack a systemic perspective and involvement of families. In the studies that did involve family members in treatment of adults, empirical evidence shows that this was beneficial (e.g. [37])

Recently, interventions for mothers with BPD to help them raise their children have been designed and are currently evaluated (e.g., [41]).

It was argued that within the professional field we should not only train specialists in the treatment of BPD but also focus on training generalists such as nurses and general practitioners to better recognize and support people with BPD. Fostering peer support programs might be another solution. Interventions that target the entire social system, including schools, work, family members and partners might be one road towards a future with less stigma and more integration.

The fourth topic, *gender dysphoria*, may especially reflect current societal changes in many European countries. Issues reflecting the association between personality disorders and gender dysphoria have not been researched enough yet. The lack of systematic research on gender identity and gender incongruence was also addressed in the recently published treatment guidelines for BPD in Germany [9]. The recommendation was to assess both BPD and gender issues,that both can be present at the same time and should be assessed carefully.

﻿The topic, gender dysphoria, may also reflect an increased awareness of diversity and recognition of minorities. Regarding the relatives' impression that there is a also a possible link with autism and gender dysphoria, there is a lack of research integrating the three domains: autism, gender dysphoria, and personality disorders. According to Van der Miesen and colleagues [46] numerous hypotheses have been proposed to explain the connection between gender dysphoria and Autism Spectrum Disorder (ASD). However, the majority of these hypotheses are not well-supported by empirical evidence. Meta-analytical results indicate that ASD and GD may be associated, though further research is necessary to determine the magnitude of this relationship [25].

On the fifth theme, *stigma,* raised by EE, there is research available emphasizing the stigma associated with a personality disorder diagnosis (especially BPD). However, a question raised like “when to be open about the diagnosis and when not” is very difficult to answer in general, while at the same time, it may be a highly relevant question for the individual. This question is usually dealt with in individual treatment and of course, the answer depends on the individual situation. Van Schie and colleagues [47] designed a study where users of lived experience and careers provided use of language recommendations for researchers and clinicians with the aim to reduce stigmatization. Participants recommended five ways of reframing language that provides: (1) acceptance, (2) connection, (3) empowerment, (4) gratitude, (5) hope and (6) validation (for details see [40]).

Although the current study may provide interesting information on specific areas of interest from EE perspective, we want to clarify that initially, the survey was not intended as a scientific study. This initiative originated organically as part of the preparation for EE involvement in the ESSPD conference. It therefore lacks methodological rigor, e.g. the selection of participants was not systematically done, sociodemographic and/or diagnostic information that could characterize the sample of participants were not systematically collected and we did not use specific qualitative research methods for analyzing data. Participating EE came from different European countries, at this point one cannot generalize the results to other countries and cultures.

Involvement of EE in research raises various challenges. In their paper “Designed to Clash?” Beeker et al [4] provide an excellent insight into the process of conducting truly collaborative research reflecting on the practical, personal, and structural challenges of collaborative research. It has been stated that there is a fundamental conflict in PPI between the researchers' attempt to pursue a scientific career, and the often very personal and idealistic motives of EE to effect change in care [12]. In addition, professionals may have an implicit desire to maintain a dichotomy between EE and themselves to not be challenged by their own experiences with mental health problems [5]. The role of emotions is another dividing factor between EE and professionals*.* The ability to hold a neutral and objective perspective is seen as a goal and sign of maturity in the scientific community – and it is usually a privilege of those who are not personally involved and affected [23].

We compared the current list of topics in research on personality disorders to the list of the JLA with TOP 10 priorities for research on depression (2016, https://www.jla.nihr.ac.uk/priority-setting-partnerships/depression/top-10-priorities/). The JLA list includes questions like: *What are the best early interventions for depression? And how early should they be used in order to result in the best patient outcomes? What is the impact on a child of having a parent with depression and can a parent prevent their child from also developing depression? What are the barriers and enablers for people accessing care/treatment when they are depressed, including when feeling suicidal, and how can these be addressed? Does depression impact employment?* Interestingly, there is a strong overlap between these questions and the topics raised for personality disorders in this report.

Currently, there are some initiatives to foster collaboration between researchers, clinicians and EE. A more practical initiative in Switzerland, Austria and Germany is the EX-IN program [18]. The organization trains people with lived experience of mental illness/crisis to become qualified peer supporters in psychiatric settings. On a national level, participation of EE in research differs between countries and, to our knowledge, there are very few initiatives specifically for personality disorders.

Thus, there may be common themes from the perspective of EE with different mental disorders and their relatives. At the same time, initiatives by EE focusing on issues of personality disorders are still rare and may be needed at a national level.

## Conclusions

Taken together, we derived five common themes – 1. prevention, early detection and intervention, 2. recovery beyond symptom improvement, 3. gender dysphoria, 4. involvement of relatives in treatment and 5. stigma-, considered to be important by EE and relatives for future research in the field of personality disorders. Of course, the field needs to continue hypotheses driven research. Ideally, from the very first steps, this research is already planned in a dialogue with EE. In our opinion, this report is an important first step to develop a future integrative research agenda, which can then be used to inform researchers, clinicians and experts with lived experience on how to improve PD research so that it matters for those who live with it. Furthermore, the initiative of the ESSPD led to the conclusion that the research field also needs to improve the communication of results to the public.

## Data Availability

Not applicable.
